# Clinicopathological Characteristics, Treatment, and Survival in Patients Diagnosed With Proximal-Type Epithelioid Sarcoma: A Case Report and Systematic Review

**DOI:** 10.7759/cureus.32962

**Published:** 2022-12-26

**Authors:** Elizabeth Zegarra Buitron, Daniel A Vidal Panduro, Domingo Morales Luna

**Affiliations:** 1 Internal Medicine, School of Medicine, Universidad Peruana de Ciencias Aplicadas, Lima, PER; 2 Pathology, Hospital Nacional Hipólito Unanue, Lima, PER

**Keywords:** proximal type, mediastinal metastasis, sarcoma, diagnosis, epithelioid sarcoma

## Abstract

Epithelioid sarcoma is a rare entity that shows a predilection for the young and middle-aged population. There are two subtypes, i.e., the distal or conventional type, which is located in distal extremities, and the proximal type, located in proximal areas of the extremities, pelvis, perineal, and genital region. The latter is characterized by more aggressive behavior, a higher recurrence rate, and poor prognosis. Histopathological and immunohistochemical diagnoses are key to correct and timely treatment and a higher survival rate. We report a case of a 41-year-old man who presented a palpable progressive growth mass in the hypogastrium. The disease time was of nine months, and the tumor was resected, but it recurred a few months later in the same location. Computed tomography (CT) scans showed images suggestive of lung metastasis and the patient had to undergo a second surgery. He received eight cycles of chemotherapy and a subsequent CT scan control showed the progression of the disease, so a new chemotherapy regimen was established. The patient received three cycles of chemotherapy without improvement, so he decided to discontinue treatment. His last outpatient medical consultation was in January 2022. A systematic review of the studies published in PubMed and Google Scholar was performed. We identified 291 articles, but only 41 reports and case series were included, with a total of 55 patients. It is important to include this type of tumor in the differential diagnosis of epithelial tumors due to its aggressive behavior. Correct and timely diagnosis is crucial to obtain lower recurrence rates, lower mortality, and higher survival rates in these patients.

## Introduction

Epithelioid sarcoma is a rare mesenchymal-origin tumor [1] present in less than 1% of patients with soft tissue tumors [2-5]. It was first described in 1970 by Ezinger [6]. According to the Surveillance, Epidemiology, and End Results (SEER) program, the incidence rate is 0.03/100,000 in the European Union and 0.05/10,000 in the United States (US), with a higher incidence in males (2:1) [7]. This tumor is divided into two histological subtypes, i.e., distal or conventional, which is characterized by being more frequent in adolescents and young adults and is located in distal extremities, and the proximal subtype, described by Guillou in 1997 [8], is more frequent in middle-aged and elderly adults, located in proximal areas of the extremities, pelvis, perineal, and genital region [5,8-11].

The proximal subtype presents a more aggressive behavior, has higher rates of recurrence and metastasis, and worse prognosis and mortality [5,8-11]. Diagnosis is made through histological and immunohistochemical analysis, which shows epithelioid-type cells, rhabdoid, or a mixed phenotype, and the presence of immunoreactivity for cytokeratins, vimentin, and epithelial markers [5,12,13]. About 90% of these tumors, in both subtypes, show loss of nuclear expression of integrase interactor 1 (INI1) [12,14]. We report a case of proximal-type epithelioid sarcoma in a 41-year-old man and elaborate a review of the clinicopathological characteristics, treatment, survival, and prognosis of this type of tumor.

## Case presentation

**Figure 1:**
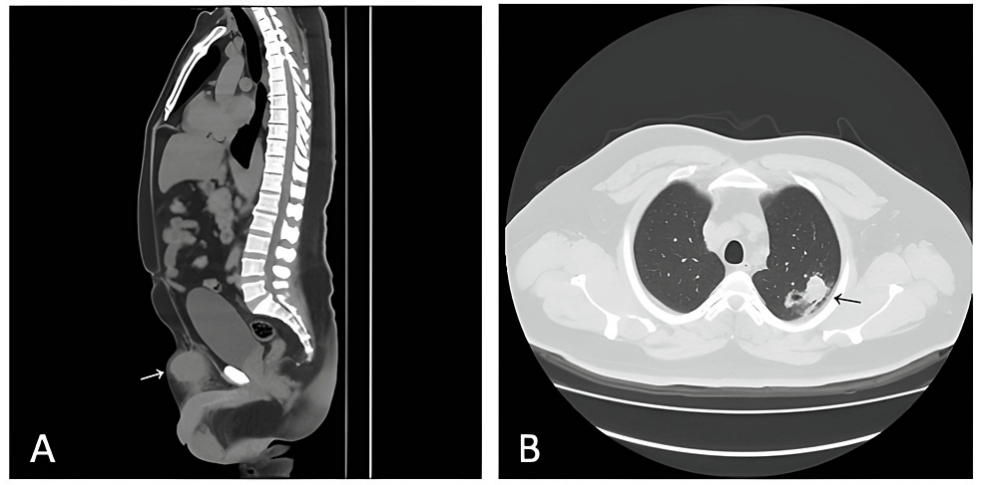
(A) Abdominopelvic CT scan with contrast, axial section: hypervascularized mass (white arrow) in subcutaneous cellular tissue in the pubic and suprapubic region suggestive of a neoplasm. (B) Chest CT scan with contrast, sagittal section: image (black arrow) suggestive of metastasis in the left lung parenchyma.

A 41-year-old male with no relevant history presented with a palpable, progressive growth of mass in the hypogastric region for nine months before admission, which was resected in another hospital. However, he did not receive adequate follow-up due to the coronavirus disease 2019 (COVID-19) pandemic. Subsequently, five months before his admission, the mass recurred in the same location, and three months later, it became painful, so he went to the emergency room. A month before his admission, the patient received the diagnosis of the first resected tumor corresponding to a malignant tumor of mesenchymal origin, for which he went to the oncology outpatient clinic of our hospital. Physical examination revealed a mass of approximately 10 cm in diameter in the hypogastric region with extension to the left inguinal region, slightly mobile, painful on palpation, and with no skin changes. No abnormalities were found in the rest of the physical examination. Laboratory tests were within normal ranges. Abdominal and pelvic computed tomography (CT) scans revealed a hypervascularized mass in subcutaneous tissue in the left pubic and suprapubic region, suggestive of a malignant neoplasm, associated with bilateral inguinal lymph nodes (Figure 1A). The chest tomography revealed a nodule in the left lung, suggestive of metastasis and the presence of mediastinal lymph node involvement (Figure 1B).

**Figure 2:**
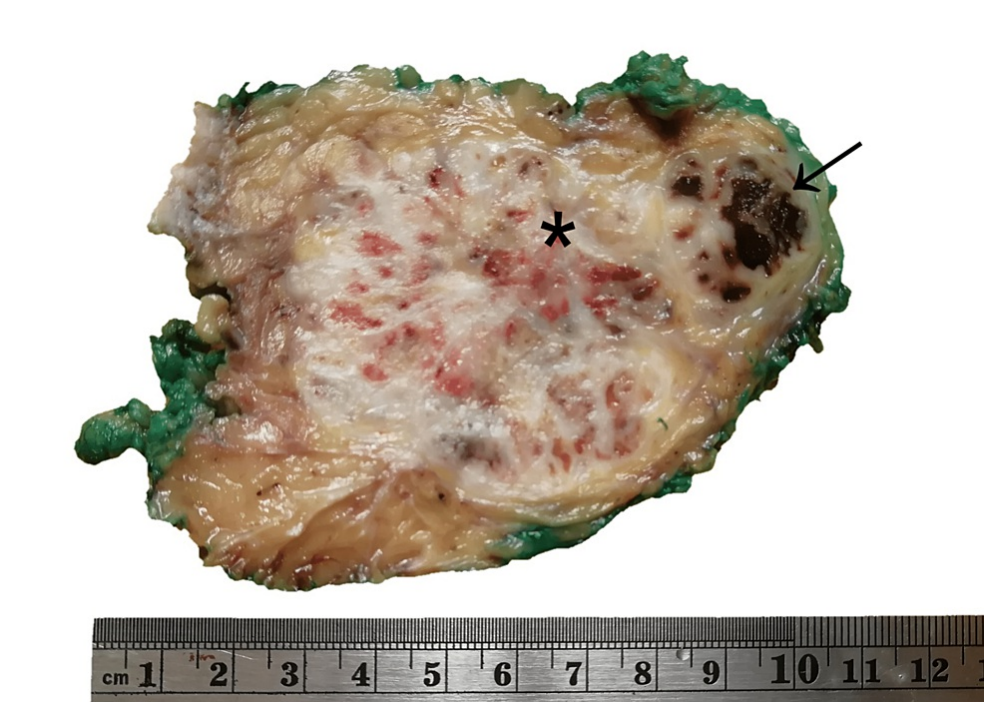
Excision of abdominal wall tumor measuring 10 x 8.5 x 7 cm, with a yellowish, multilobular cut surface with cystic areas with a necrotic (black arrow) and hemorrhagic (asterisk) appearance.

**Figure 3:**
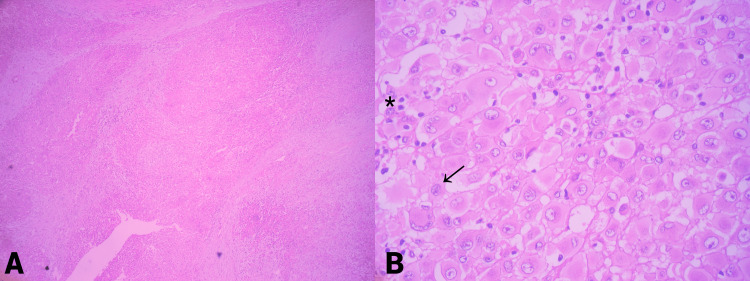
Hematoxylin and eosin (H&E) staining. (A) H&E x 40: infiltrative pattern with the formation of large oval nodules. (B) H&E x 400: large, polygonal cells with defined borders, with dense eosinophilic cytoplasm and epithelial appearance. The nuclei are large, vesicular, with prominent nucleoli (black arrow). Some cells have a rhabdoid appearance and others are multinucleated (asterisk).

**Figure 4:**
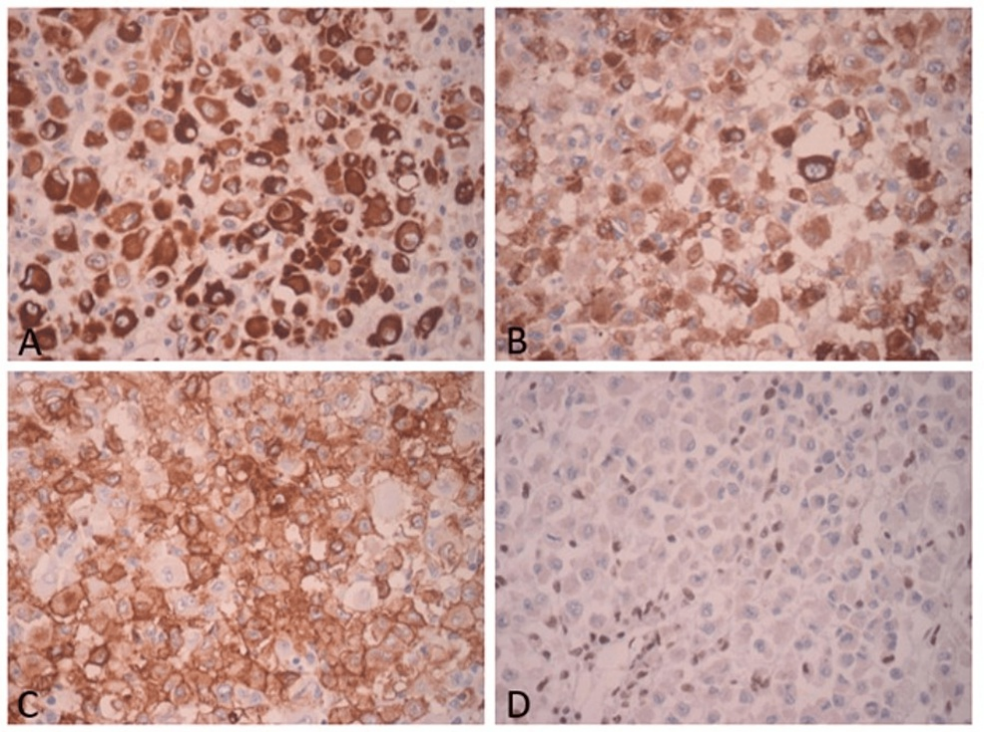
Neoplastic cells show immunoexpression for (A) PANCK - AE1/AE3, (B) EMA, (C) CD34, and (D) loss of nuclear expression in malignant cells for INI1. PANCK: pancytokeratin; EMA: epithelial membrane antigen; INI1: integrase interactor 1.

The patient underwent surgical resection of the tumor with wide margins. Post-surgical evolution showed no complications. The resected tumor measured 10 x 8.5 x 7 cm, and had a yellowish cut surface, with a lobular appearance with cystic areas, the largest being 1 x 0.8 x 0.8 cm. The presence of hemorrhagic and necrotic areas was identified (Figure 2). The histological examination showed multinodular growth with areas of tumor necrosis, comprised of epithelioid-appearing cells with eccentric nuclei, and an ample and eosinophilic cytoplasm; some cells had a rhabdoid appearance with abundant mitotic figures. Likewise, the lymphovascular invasion was identified. The surgical margins were free of tumors. These findings were consistent with proximal-type epithelioid sarcoma (Figure 3). Immunohistochemical analysis showed reactivity for pancytokeratin, epithelial membrane antigen (EMA), CD34, and loss of nuclear expression for INI1 (Figure 4).

The patient received eight cycles of chemotherapy with gemcitabine (1800 mg) and docetaxel (200 mg) for nine months. A control chest CT scan was performed 10 months after surgery, showing the progression of the disease in the lungs. A second regimen with doxorubicin was performed, which continued for three cycles, but since there was no improvement, the patient refused to continue the treatment. The last follow-up by oncology was in January 2022, but there is no updated information on the patient.

## Discussion

**Figure 5:**
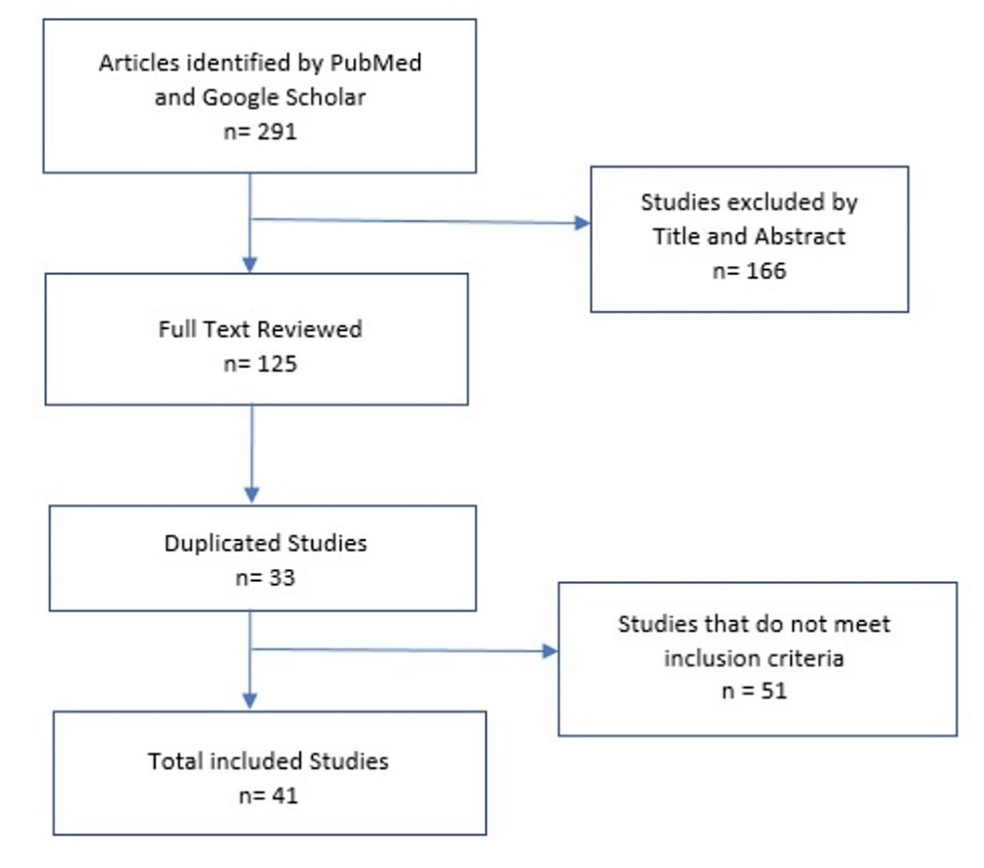
Item selection flowchart.

At first, 291 studies were identified, and they were filtered by titles and abstracts, which gave us 125 full-text studies to be evaluated, of which 30 were duplicates. The inclusion criteria were articles that included at least one case report that described gender, age, location and size of the tumor, immunohistochemical profile, treatment, and survival. The exclusion criteria were those articles that did not meet the aforementioned inclusion criteria. Fifty-one articles did not meet the inclusion criteria. Only 41 studies were included, of which 35 were case reports and six were case series, analyzing a total of 55 cases (Figure 5).

Clinical findings

A total of 55 patients were included, in whom the age range fluctuated between nine and 87 years, of which 18 patients (32.73%) were men and 37 (67.27%) were women, and two were pregnant [15,16]. The time from the onset of signs and symptoms and diagnosis ranged from seven days to 36 months. However, 17 studies did not include this variable [10,17]. Thirty-seven patients (67.28%) presented with an initial sign of a rapidly growing palpable mass, mostly painful [3,9,18-22]. Other symptoms and signs were presented according to the affected area like headaches, neck pain, nausea, vomiting, abdominal pain, abdominal fullness, visual disturbances, palpebral ptosis, eye pain and protrusion, ectropion, epiphora, anorexia, weight loss, dyspnea, edema in the affected area, constipation, fever, rectal bleeding, vaginal bleeding, and epistaxis.

The tumor was located in different parts of the body. The most common site affected was the pelvic region, with 24 cases, followed by the adrenal gland and the orbit of the eye, with three cases each. The other locations of the tumors were described in Table 1.

**Table 1:** Comparison of cases of proximal-type epithelioid sarcoma. M: male; F: female; RT: radiotherapy; CT: chemotherapy; SX: surgery; LND: lymphadenectomy; P: pregnant; AD: active disease; R: recurrence; NR: no recurrence.

Author	Sex	Age (years)	Site	Size (cm)	Metastasis at presentation	Treatment	Follow-up period (months)	Clinical outcome	
									Echchaoui et al. (2016) [1]	F	20	Scapula	3.5	No	SX	18	NR	
Rodrigues et al. (2015) [3]	F	55	Vulva	10 x 4	Yes	Palliative care	<1	Deceased	
Gao et al. (2020) [4]	F	16	Maxillary sinus	2.5 x 3	No	SX	36	NR	
Dos Santos et al. (2013) [9]	M	25	Gluteus	4	Yes	CT	9	Deceased	
Huang et al. (2019) [10]	F	31	Adrenal gland	4.4 x 2.9 x 3.3	No	CT	2	Deceased	
Manzanares et al. (2011) [11]	F	57	Pubis	4 x 3 x 2	No	SX	96	Recurrence 4 years after SX	
Mannan et al. (2010) [12]	M	47	Inguinal region	10 x 7	No	SX	14	NR	
Gómez et al. (2015) [13]	F	11	Lumbar vertebra (L3)	>5	No	SX (surgical reconstruction), CT, RT, orthopedic fixation	6	NR	
Orita et al. (2022) [15]	F (P)	36	Vulva	5 x 3 x 1	No	SX, LND, CT	60	NR	
Moore et al. (2002) [16]	F (P)	29	Vulva	3.5	Yes	SX, CT, RT	6	Deceased	
Alikhan et al. (2016) [17]	F	27	Kidney	3.3	Yes	SX, CT, RT	31	Deceased	
	M	72	Adrenal gland	3.6	No	SX, RT	29	NR	
Liu et al. (2018) [18]	M	42	Gingival mucosa	4	No	SX, LND	3	NR	
Zhang et al. (2019 ) [19]	M	80	Scalp	1.5 x 1 x 0.8	No	SX	12	NR	
Yahiro et al. (2022) [20]	F	24	Vulva	5.3 x 3.5 x 6.3	No	SX, RT	3	Deceased	
Kaya et al. (2018) [21]	F	87	Eye orbit	2.2 x 2 x 1.2	No	SX, RT	3	NR	
Bravo-Taxa et al. (2021) [22]	F	53	Vulva	33 x 30 x 14	Yes	SX, LND, CT, RT	9	NR	
Patrizi et al. (2013) [23]	F	63	Vulva	4	Yes	SX, LND, RT	14	NR	
Ong et al. (2012) [24]	F	51	Vulva	1.1 x 1 x 1	No	SX, LND	8	NR	
Zejun et al. (2019) [25]	F	47	Intrasellar, suprasellar	4.5 x 4 x 3.5	No	SX	<1	Deceased	
	F	52	Intrasellar, suprasellar	3 x 4 x 4	No	SX	<1	Deceased	
Frank et al. (2013) [26]	M	43	Retro-orbital region	2.4 x 3.4 x 1.5	No	SX, CT	36	R	
	F	71	Nasal cavity	3.5 x 3 x 2.9	No	SX	29	NR	
Al-Salam et al. (2010) [27]	F	9	Submandibular region	4 x 3 x 3	No	SX	12	NR	
Sakurai et al. (2018) [28]	F	47	Costal wall	7.5	No	SX	22	NR	
Kim et al. (2008) [29]	F	24	Vulva	2 x 1.5	No	SX	8	NR	
Yue et al. (2018) [30]	F	41	Pubis	2	No	SX	9	NR	
Chan et al. (2016) [31]	F	32	Vulva	1.5 x 0.5 x 0.5	No	SX	9	NR	
Monappa et al. (2022) [32]	M	11	Adrenal gland	10.8 x 10.8 x 13.5	No	SX	6	NR	
Li et al. (2019) [33]	M	48	Thigh	6	Yes	SX (amputation)	38	NR	
	F	40	Chest wall	7	Yes	SX	6	NR	
	F	26	Tongue	0.5	No	SX	23	NR	
	M	54	Inguinal region	10	Yes	Treatment refusal	15	NR	
	M	39	Lungs	2	Yes	CT	20	NR	
	M	47	Thigh	3	Yes	CT	30	NR	
	M	74	Lungs	15	Yes	SX	12	NR	
	F	63	Vertebral body	2	Yes	Treatment refusal	14	NR	
Babu et al. (2013) [34]	F	25	Cervical region	5 x 5.8 x 5.1	Yes	SX (laminectomy)	-	Deceased post-SX	
Fukunaga et al. (1999) [35]	M	46	Pelvis	8 x 6 x 6	Yes	SX, LND	5	Deceased	
Dash et al. (2022) [36]	F	45	Vulva	2 x 2	No	SX, RT	66	R	
	F	27	Vulva	3 x 2	No	SX, LND, RT	24	NR	
	F	37	Vulva	7	Yes	SX, LND	12	NR	
	F	25	Vulva	8 x 5	Yes	RT, CT	2	AD	
Kasamatsu et al. (2001) [37]	F	23	Vulva	5.5 x 2.5	Yes	SX, LND	6	NR	
Kim et al. (2012) [38]	F	41	Mons pubis	8	No	SX, RT	10	NR	
Nardone et al. (2015) [39]	M	32	Lumbar region	4.5 x 4.7	No	SX (laminectomy) RT	13	Deceased	
	M	50	Eye orbit	2 x 1	No	SX, RT	48	NR	
Gambini et al. (2004) [40]	F	12	Gluteus	4.5	No	SX, CT, RT	8	NR	
Magetsari et al. (2020) [41]	M	39	Femur (proximal region)	12.2 x 9.8 x 13.8	No	SX (hip disarticulation), CT, RT	12	NR	
Del Pozo et al. (2002) [42]	M	25	Costal wall	3.5	No	SX, RT, CT	7	NR	
Argenta et al. (2007) [43]	F	35	Vulva	4 x 3	No	SX, LND, RT	40	NR	
Jeon et al. (2020) [44]	F	77	Vulva	3 x 2	Yes	SX, LND, RT	36	AD	
Chung et al. (2021) [45]	F	24	Vulva, abdomen, anus, inguinal region	12 x 7 x 8	Yes	RT, CT	19	Deceased	
Gutiérrez et al. (2004) [46]	M	22	Nape	12 x 10	Yes	RT,CT	18	Deceased	
Altundag et al. (2004) [47]	F	51	Vulva	3.5 x 2.5	Yes	SX, LND, CT, RT	6	Deceased	

The form of presentation of each lesion varied even when it was in the same region. In the vulva, the presentation of each lesion was different; in one of the cases, it presented as an ulcerated and indurated lesion [23], while in other cases, the presence of painless masses of variable growth was observed [3,24]. The size of the tumors was variable according to the location, with a minimum of 1.1 cm to a maximum of 33 cm [22,24].

Regarding treatment, 19 patients were treated surgically by wide local excision [1,4,11,12,19,25-34], five with surgery plus lymphadenectomy [18,24,35-37], seven underwent surgery and adjuvant radiotherapy [17,20,21,36,38,39], one underwent surgery and chemotherapy [26], six had surgery, chemotherapy, and adjuvant radiotherapy [13,16,17,40-42], four underwent surgery, lymphadenectomy, and radiotherapy [23,36,43,44], one had surgery, lymphadenectomy, and chemotherapy [15], two underwent surgery, lymphadenectomy, radiotherapy, and chemotherapy, three received palliative radiotherapy and chemotherapy [36,45,46], and four underwent treatment with chemotherapy alone [9,10,33]. However, one patient, with metastases at presentation, received only palliative care [3] and two patients refused treatment [33].

Pathological findings

Histologically, most cases were characterized by oval, polygonal, and pleomorphic epithelioid cells with abundant eosinophilic cytoplasm and enlarged vesicular nuclei with prominent nucleoli. In 22 cases, rhabdoid-looking areas were reported [4,9,11,13,17,20,21,25,34-36,38-42,46,47].

The presence of hemorrhage [15,17,22,33,40,42] and necrosis [10,15-19,21-23,25-27,32-34,36,37,40-42,47] were common findings present in eight and 23 cases, respectively. High mitotic activity was reported in 18 tumors [3,4,10,12,16,18,19,22,24,25,31,32,35,37,40-42], and in four of them, more than 20 mitoses were observed per 10 high-power fields [4,22,35,40]. Vascular invasion was reported in four cases [22,32,36]. Other histologic features were spindle cell proliferation (14 cases) [16,19,22,23,26,27,29,30,32,33,35,37,41,42], presence of signet ring cells (one case) [4], anaplasia (two cases) [23,47], and pseudoangiosarcomatous features (one case) [35]. Proximal epithelioid sarcoma was the initial diagnosis in most patients. However, in five cases, other diagnoses were initially established, such as adrenal cortical carcinoma [10], squamous cell carcinoma [4], fibroma [36], intermediate-grade epithelioid carcinoma [21], and dermal necrosis [37].

Immunohistochemical findings

Immunohistochemical results are summarized in Table 2. Forty-one cases (73.21%) presented immunoreactivity for vimentin and 50 cases (90.90%) for cytokeratin. The epithelial membrane antigen (EMA) was positive in 35 tumors (63.63%). A total of 28 tumors (50.90%) tested positive for CD34. Likewise, Ki-67 was positive in 13 tumors with a proliferation ranging from 10% to 80%. Immunoreactivity for muscle markers, desmin, was positive in only two tumors and alpha-smooth muscle actin (SMA) was positive in only five tumors. The reactivity of CD31 was analyzed in 17 tumors, and 14 of them were negative (82.35%). Ten cases (17.85%) were positive for other markers such as S-100 protein, neuron-specific enolase (NSE), synaptophysin, CD56, and p53. The analysis for detection of INI1 showed loss of its expression in the nucleus of tumor cells in 24 cases (44.63%). In three of these cases, fluorescence in situ hybridization (FISH) was negative [17,25].

**Table 2:** Proximal-type epithelioid sarcoma immunohistochemical characteristics. VIM: vimentin; CK: cytokeratin; EMA: epithelial membrane antigen; DES: desmin; SMA: smooth muscle actin; INI: integrase interactor; SMARCB1: SWI/SNF-related matrix-associated actin-dependent regulator of chromatin, subfamily B-member 1; FISH: fluorescence in situ hybridization; LE: loss of expression.

Case report	VIM	CK	EMA	DES	SMA	CD34	CD31	KI67	INI/SMARCB1	FISH
Echchaoui et al. (2016) [1]	+	+				+				
Rodrigues et al. (2015) [3]	+			−	−	−				
Gao et al. (2020) [4]		+					−		LE	
Dos Santos et al. (2013) [9]	+	+	+			+				
Huang et al. (2019) [10]		+		−		+	−		LE	
Manzanares et al. (2011) [11]	+	+	+		−	+		20%		
Mannan et al. (2010) [12]	+	+	+	−	−	+			LE	
Gómez et al. (2015) [13]	+	+	+							
Orita et al. (2022) [15]	+	+		−		+			LE	
Moore et al. (2002) [16]	+	+	+							
Alikhan et al. (2016) [17]		+	+			+	+		LE	
		+	+			+	+		LE	−
Liu et al. (2018) [18]	+	+			+		+	60-70%		
Zhang et al. (2019 ) [19]	+	+		−	−	−		15%		
Yahiro et al. (2022) [20]		+	+			+			LE	
Kaya et al. (2018) [21]	+	+	+	−					LE	
Bravo-Taxa et al. (2021) [22]	+	+	+	−	+	−			LE	
Patrizi et al. (2013) [23]	+	+	−	−	+	−		+		
Ong et al. (2012) [24]	+	−	+	−		+			LE	
Zejun et al. (2019) [25]	+	+	+	−	−	+			LE	−
	+	+	+	−	−	+			LE	−
Frank et al. (2013) [26]		+		+						
			+	−					LE	
Al-Salam et al. (2010) [27]	+	+	+	−	−	+	−			
Sakurai et al. (2018) [28]		+				+	−		LE	
Kim et al. (2008) [29]	+	+								
Yue et al. (2018) [30]		−	+	−	−	−	−	15%	LE	
Chan et al. (2016) [31]		+			+				LE	
Monappa et al. (2022) [32]	+	+				+			LE	
Li et al. (2019) [33]	+	+	+	−	−	−	−	80%		
	+	+	+		−	−		60%		
	+	+	+		−	+	−	10%		
	+	+			−	−	−	80%		
	+	−	+	+	−					
	+	+		−	−	+		30%		
	+	+	+	−		−		20%		
	+	+	−			−	−	20%		
Babu et al. (2013) [34]	+	+	+	−	−	−		50%	LE	
Fukunaga et al. (1999) [35]	+	+	+	−	−	+	−			
Dash et al. (2022) [36]		+				+			LE	
		+	+			+			LE	
		+	+	−					LE	
		+	+			+			LE	
Kasamatsu et al. (2001) [37]	+	+	+	−	+	+	−			
Kim et al. (2012) [38]	+	+	+	−		+			LE	
Nardone et al. (2015) [39]	+	+	+		−	+	−			
	+	+	+		−	+	−			
Gambini et al. (2004) [40]	+	+	+	−	−	+	−			
Magetsari et al. (2020) [41]	+	+				−				
Del Pozo et al. (2002) [42]	+	+		−						
Argenta et al. (2007) [43]	+	+	+			−				
Jeon et al. (2020) [44]	+	+				+				
Chung et al. (2021) [45]	+	+	+	−					LE	
Gutiérrez et al. (2004) [46]	+	+	+							
Altundag et al. (2004) [47]	+	+	+	−	−	+				

Monitoring and forecast

All the included patients had follow-up data. Six patients (10.71%) developed local recurrence in the same place. The period between the primary excision and the first local recurrence ranged from two months to four years. A total of 22 patients (40%) developed metastases at the beginning of the diagnosis: 12 patients in regional and distant lymph nodes, 11 in the lung, four in the liver, three in the brain, three in the bone, one in the adrenal gland, one in the ovary, one in the sacrum, one in the scalp and back, one in the mediastinum, one in the kidney, and one in the calf. In the last follow-up, 14 patients (25%) had died due to the disease. Nine of them, with metastasis at the beginning of the diagnosis, died in a period between one day and 31 months after surgery; four patients without metastasis at the beginning of the diagnosis died in a shorter time, from one month to 13 months after treatment. A total of 42 patients (75%) had no evidence of disease in a follow-up period of up to five years.

Epithelioid sarcoma is a rare tumor that occurs more frequently in adolescents and adults (mean age: 35 years) with a predominance in males [5,40,47]. They are classified into two types: distal or conventional, located in superficial areas of the upper and lower extremities and with greater involvement of young adults; and the proximal type, located in proximal areas such as the trunk, abdomen, pelvis, perineum, and genitalia; with greater affectation to young and middle-aged adults [5,10,11]. The latter presents the most aggressive behavior, with frequent recurrences, early metastasis, and a worse prognosis [3,5,9,24,40].

In most cases, the tumor presents as single or multiple nodules with slow, progressive, and painless growth [1,11,12,16,24,27-30,32,33,36-40,44-47]. However, it can sometimes be accompanied by superficial ulceration, hemorrhage, necrosis, and plaques [3,10,11,18]. The growth period can vary from six months to five years [1], and it can be mistaken for a benign tumor, which can delay an early diagnosis and treatment, consequently worsening the prognosis [1,3]. Regional spread through the lymphatic drainage or direct extension more often affects regional lymph nodes, while distant metastasis due to hematogenous spread more often affects the lung and liver [3,11,24].

Rodrigues et al. [3] describe these lesions as a nodule or solid white masses. Likewise, if it presents necrosis or hemorrhage, it turns yellowish or brown, respectively [3,18,48]. Histologically, it is mainly characterized by epithelioid cells with different degrees of pleomorphism, eosinophilic cytoplasm, enlarged vesicular nuclei, and prominent nucleoli [5,18]. Also, it frequently presents rhabdoid differentiation, cellular atypia, and the absence of a granulomatous pattern [5,9,11,25,35,40]. Occasionally, it can present pseudoangiomatous architecture and spindle cells [12,15]. The presence of foci of necrosis and mitotic activity, which is usually low (less than five per high-power field), are unfavorable histological features [3,10,24].

The immunohistochemical study is of vital importance to differentiate this neoplasm from other tumors with an epithelioid appearance [3,47]. It is characterized by immunoreactivity for epithelial markers, such as cytokeratins and EMA, and mesenchymal markers such as vimentin and CD34 in half of the cases [1,3,11,25]; it is usually negative for S-100 protein, CD31, and desmin [3,10,18,47]. The differential diagnosis includes sarcomatoid carcinoma, which differs in that it does not show reactivity to CD34 [47]; similarly, synovial sarcoma [4,48], squamous cell carcinoma (vimentin and desmin negative), malignant melanoma (S-100 and HMB positive), epithelioid angiosarcoma (CD31 positive), rhabdomyosarcoma (desmin and cytokeratin negative), epithelioid leiomyosarcoma (actin and desmin positive) [3], malignant rhabdoid tumors (CD34 negative) [4], osteosarcoma (cytokeratin and CD34 negative) [4], and undifferentiated carcinoma (CD34 negative) [48]. The ERG marker, present in tumors of endothelial origin such as angiosarcomas, hemangiomas, and hemangioendothelioma, can be present in epithelioid sarcoma. Therefore, it could be a potential diagnostic pitfall in this context [49].

INI1 (Hsnf5/SMARCB1) is a member of the SWI/SNF (SWItch/Sucrose Non-Fermentable) chromatin remodeling complex, located on chromosome 22q11.2 [14,25]. It is found ubiquitously in normal tissues and functions as a tumor suppressor gene by controlling the cell cycle and regulating the cytoskeleton [5,14,17,25]. Mutations and deletions in the SMARCB1 gene were also reported in rhabdoid tumors of the kidney and the central nervous system [17].

However, later studies have shown the loss of nuclear expression of SMARCB1/INI1 in other types of tumors, such as renal medulla carcinoma (100%), malignant peripheral nerve sheath tumor (50%), myoepithelial tumors, extraskeletal myxoid chondrosarcomas, sinonasal carcinoma, and, more recently, epithelioid sarcoma [5,10,17,25]. Hornick et al. carried out a study in which 93% of epithelioid sarcomas presented a complete loss of INI1 expression, among these, 91% were distal and 95% were proximal [14]. Therefore, this study can help confirm the diagnosis of this type of tumor.

The treatment of choice for this type of tumor is local excision with wide margins, at least 2 cm [1,11,18,24,47]. Adjuvant radiotherapy is recommended in high-grade sarcomas or those with positive margins [1,3,10,11,24]. Also, the combination of surgery and radiotherapy decreases local recurrence [4,5,11,47]. However, 40-60% of patients with high-grade sarcoma will die of metastatic disease [23,47]. So far, no beneficial effects of locoregional lymphadenectomy on local or distant relapse rates have been demonstrated [3,23,24,47]. According to studies, it is indicated if the lymphadenopathies are suspicious for malignancy or show an increase in size [23,47]. The benefits of adjuvant chemotherapy are not yet established [5,11,23], but it could be recommended in cases of metastatic disease [1,3].

This tumor is characterized by aggressive behavior and poor prognosis [8,41,43,45,46,48]. According to studies, poor prognostic factors are regional lymph node involvement, early metastasis, high-grade tumors, vascular invasion, tumor size greater than 2 cm, deep location, and presence of necrosis [3,18,47].

The range of local recurrence was 34-77% [10,18], and in most cases, it developed within six months after the start of treatment [47], and of these, 60-75% developed metastases, mainly to regional lymph nodes and lung [3-5,11], with a survival rate between eight to 10 months [18]. The overall survival rate for the proximal type is 31.3%, compared to the distal type (90.2%) [11,12]. Hasegawa et al. evaluated 20 patients with proximal-type epithelioid carcinoma, of which 65% developed local recurrence and 75% had metastasis, mainly to lymph nodes and lung; 65% of the patients died from the disease [48].

## Conclusions

We report a case of proximal-type epithelioid sarcoma in the hypogastrium and describe the clinicopathological characteristics, treatment, survival, and prognosis of this pathology. This type of tumor is rare but aggressive and with a poor prognosis. However, it can be confused with benign or less aggressive epithelioid lesions. Pathologic analysis is characterized by the presence of large polygonal cells, prominent nucleoli and areas of necrosis, and immunoreactivity for cytokeratins. On the other hand, the immunohistochemical profile is characterized by the presence of epithelial markers and the loss of INI1 expression. These studies are essential for a correct and timely diagnosis, to establish the appropriate and early treatment with the aim of obtaining a lower recurrence rate and a higher survival rate among these patients.

## References

[1] Echchaoui A, Sadrati Y, Elbir Y (2016). Proximal-type epithelioid sarcoma: a new case report and literature review. Pan Afr Med J.

[2] Czarnecka AM, Sobczuk P, Kostrzanowski M, Spalek M, Chojnacka M, Szumera-Cieckiewicz A, Rutkowski P (2020). Epithelioid sarcoma—from genetics to clinical practice. Cancers (Basel).

[3] Rodrigues AI, Lopes HI, Lima O, Marta S (2015). Proximal-type epithelioid sarcoma—unusual presentation: unilateral vulvar mass. BMJ Case Rep.

[4] Gao W, Xue J, Ma C, Hu J (2020). Proximal-type epithelioid sarcoma in the maxillary sinus: an unusual presentation. Int J Clin Exp Pathol.

[5] Thway K, Jones RL, Noujaim J, Fisher C (2016). Epithelioid sarcoma: diagnostic features and genetics. Adv Anat Pathol.

[6] Ezinger FM (1970). Epithelioid sarcoma. A sarcoma simulating a granuloma or a carcinoma. Cancer.

[7] Frezza AM, Botta L, Pasquali S (2017). An epidemiological insight into epithelioid sarcoma (ES): the open issue of distal-type (DES) versus proximal-type (PES). Ann Oncol.

[8] Guillou L, Wadden C, Coindre JM, Krausz T, Fletcher CD (1997). "Proximal-type" epithelioid sarcoma, a distinctive aggressive neoplasm showing rhabdoid features. Clinicopathologic, immunohistochemical, and ultrastructural study of a series. Am J Surg Pathol.

[9] Santos LM, Nogueira L, Matsuo CY, Talhari C, Santos M (2013). Proximal-type epithelioid sarcoma - case report. An Bras Dermatol.

[10] Huang X, Nayar R, Zhou H (2019). Primary adrenal gland epithelioid sarcoma: a case report and literature review. Diagn Cytopathol.

[11] Manzanares-Campillo MC, Muñoz-Atienza V, Sanchez-Garcia S, Gil-Rendo A, Jara-Sanchez A, Fernandez JM (2011). Sarcoma epitelioide de tipo proximal en pubis. Presentación de un caso y revisión de la literatura. Cir Cir.

[12] Mannan AA, Rifaat AA, Kahvic M (2010). Proximal-type epithelioid sarcoma in the groin presenting as a diagnostic dilemma. Pathol Oncol Res.

[13] Gómez García WC, Friedrich P (2015). Primary non-metastatic epithelioid sarcoma of lumbar vertebra L3: pediatric case report and brief review of the literature. J Tumor.

[14] Hornick JL, Dal Cin P, Fletcher CD (2009). Loss of INI1 expression is characteristic of both conventional and proximal-type epithelioid sarcoma. Am J Surg Pathol.

[15] Orita Y, Kamio M, Tokudome A, Kitazono I, Ichihara F, Kobayashi H (2022). Successful management of vulvar proximal-type epithelioid sarcoma in pregnancy. Gynecol Oncol Rep.

[16] Moore RG, Steinhoff MM, Granai CO, DeMars LR (2002). Vulvar epithelioid sarcoma in pregnancy. Gynecol Oncol.

[17] Alikhan MB, Pease G, Watkin W, Grogan R, Krausz T, Antic T (2017). Primary epithelioid sarcoma of the kidney and adrenal gland: report of 2 cases with immunohistochemical and molecular cytogenetic studies. Hum Pathol.

[18] Liu Y, Sun B, Yang Y (2018). Proximal-type epithelioid sarcoma of the oral cavity: a case report and literature review. Oral Surg Oral Med Oral Pathol Oral Radiol.

[19] Zhang XW, Deng YJ, Zhou L, Deng H (2019). Epithelioid sarcoma of the scalp: a case report and literature review. Int J Clin Exp Pathol.

[20] Yahiro S, Fujimoto T, Fujita I (2022). Proximal-type epithelioid sarcoma in pubic region expressing L-type amino acid transporter 1: a case report. SAGE Open Med Case Rep.

[21] Kaya EA, Broadbent TJ, Thomas CJ (2018). Primary epithelioid sarcoma of orbit: a case report and review of the literature. Case Rep Oncol Med.

[22] Bravo-Taxa M, Taxa-Rojas L, López-Blanco A (2021). Proximal epithelioid sarcoma of the vulva: a case report and review of the literature. Clin Surg Res Commun.

[23] Patrizi L, Corrado G, Saltari M, Perracchio L, Scelzo C, Piccione E, Vizza E (2013). Vulvar "proximal-type" epithelioid sarcoma: report of a case and review of the literature. Diagn Pathol.

[24] Ong AC, Lim TY, Tan TC, Wang S, Raju GC (2012). Proximal epithelioid sarcoma of the vulva: a case report and review of current medical literature. J Obstet Gynaecol Res.

[25] Zejun D, Kun Y, Dehong L, Xueling Q (2019). Proximal-type epithelioid sarcoma in skull base: a pathological diagnosis challenge with other intracranial tumors. Pathol Oncol Res.

[26] Frank R, Sadri N, Bhatti T, Biegel JA, Livolsi VA, Zhang PJ (2013). Proximal-type epithelioid sarcoma of the head and neck (HN): a study with immunohistochemical and molecular analysis of SMARCB1. J Clin Exp Oncol.

[27] Al-Salam S, Al Ashari M (2010). Epithelioid sarcoma in a child presenting as a submandibular mass. Afr Health Sci.

[28] Sakurai T, Kusumoto H, Wakasa T, Ohta Y, Konishi E, Shiono H (2018). Epithelioid sarcoma in the chest wall: a case report and literature review. Surg Case Rep.

[29] Kim JH, Choi YS, Lee TS (2008). A case of epithelioid sarcoma arising in the vulva. J Gynecol Oncol.

[30] Yue Y, Lu Y, Ma X, Tang Z, Cheng Y (2018). Ultrasonography findings of proximal-type epithelioid sarcoma of the vulva: a case report. Mol Clin Oncol.

[31] Han CH, Li X, Khanna N (2016). Epithelioid sarcoma of the vulva and its clinical implication: a case report and review of the literature. Gynecol Oncol Rep.

[32] Monappa V, Singh VK, Chawla A (2022). Primary adrenal epithelioid sarcoma in a child: a case report with literature review. Fetal Pediatr Pathol.

[33] Li Y, Cao G, Tao X, Guo J, Wu S, Tao Y (2019). Clinicopathologic features of epithelioid sarcoma: report of seventeen cases and review of literature. Int J Clin Exp Pathol.

[34] Babu R, Karikari IO, Cummings TJ, Gottfried ON, Bagley CA (2013). Treatment and outcomes of epithelioid sarcoma of the spine. J Clin Neurosci.

[35] Fukunaga M, Ushigome S (1999). Proximal-type epithelioid sarcoma in the pelvic soft tissues. APMIS.

[36] Dash B, Rekhi B, Shylasree TS, Maheshwari A, Bajpai J (2022). Proximal-type epithelioid sarcoma of vulva - case series of a rare tumor. Gynecol Oncol Rep.

[37] Kasamatsu T, Hasegawa T, Tsuda H (2001). Primary epithelioid sarcoma of the vulva. Int J Gynecol Cancer.

[38] Kim HJ, Kim MH, Kwon J, Kim JY, Park K, Ro JY (2012). Proximal-type epithelioid sarcoma of the vulva with INI1 diagnostic utility. Ann Diagn Pathol.

[39] Nardone A, Anichini G, Caporlingua F, Antonelli M, Lapadula M, Santoro A, Salvati M (2015). Proximal-type epithelioid sarcoma: report of two cases with unusual location and review of the literature. J Spine Neurosurg.

[40] Gambini C, Sementa A, Rongioletti F (2004). "Proximal-type" epithelioid sarcoma in a young girl. Pediatr Dermatol.

[41] Magetsari R, Dwianingsih EK, Putro YA, Araneta I, Sakti YM (2020). Primary rhabdoid epithelioid sarcoma of the left thigh mimicking epithelioid rhabdomyosarcoma: a diagnostic pitfall. Int J Surg Case Rep.

[42] Del Pozo M, Gorron R, Poblete MT (2002). Sarcoma epitelioide de localización proximal. Presentación de un caso y revisión de la literatura. Cuad Cir.

[43] Argenta PA, Thomas S, Chura JC (2007). Proximal-type epithelioid sarcoma vs. malignant rhabdoid tumor of the vulva: a case report, review of the literature, and an argument for consolidation. Gynecol Oncol.

[44] Jeon SE, Lee J, Jung HJ (2020). An extremely rare elderly case of proximal epithelioid sarcoma of the vulva: case report with a review of literatures. Obstet Gynecol Sci.

[45] Chung H, Jang TK, Kwon SY, Ha J, Shin SJ (2021). A proximal type epithelioid sarcoma of the vulva with multiple distant metastases: A case report and review of the literature. Gynecol Oncol Rep.

[46] Gutiérrez Díaz Ceballos MA, Aguirre Quezada DE, Trujillo de Anda AP, Cruz Ortiz H (2004). Sarcoma epitelioide de tipo proximal (lesión letal e infrecuente). Informe de un caso. Rev Med Hosp Gen Mex.

[47] Altundag K, Dikbas O, Oyan B, Usubutun A, Turker A (2004). Epithelioid sarcoma of vulva: a case report and review of the literature. Med Oncol.

[48] Hasegawa T, Matsuno Y, Shimoda T, Umeda T, Yokoyama R, Hirohashi S (2001). Proximal-type epithelioid sarcoma: a clinicopathologic study of 20 cases. Mod Pathol.

[49] Miettinen M, Wang Z, Sarlomo-Rikala M, Abdullaev Z, Pack SD, Fetsch JF (2013). ERG expression in epithelioid sarcoma: a diagnostic pitfall. Am J Surg Pathol.

